# Human Milk Oligosaccharides in Combination with Galacto- and Long-Chain Fructo-Oligosaccharides Enhance Vaccination Efficacy in a Murine Influenza Vaccination Model

**DOI:** 10.3390/nu16172858

**Published:** 2024-08-26

**Authors:** Mehrdad Azarmi, Negisa Seyed Toutounchi, Astrid Hogenkamp, Suzan Thijssen, Saskia A. Overbeek, Johan Garssen, Gert Folkerts, Belinda van’t Land, Saskia Braber

**Affiliations:** 1 Division of Pharmacology, Utrecht Institute of Pharmaceutical Science (UIPS), Utrecht University, 3584 CG Utrecht, The Netherlands; 2 Danone Global Research and Innovation Center B.V., 3584 CT Utrecht, The Netherlands; 3 Department of Pediatric Immunology, Wilhelmina Children Hospital, University Medical Center Utrecht, 3584 EA Utrecht, The Netherlands

**Keywords:** B lymphocytes, fructo-oligosaccharides, galacto-oligosaccharides, human milk oligosaccharides, prebiotics, T helper 1 immune response, vaccination

## Abstract

Early-life nutrition significantly impacts vaccination efficacy in infants, whose immune response to vaccines is weaker compared to adults. This study investigated vaccination efficacy in female C57Bl/6JOlaHsd mice (6 weeks old) fed diets with 0.7% galacto-oligosaccharides (GOS)/long-chain fructo-oligosaccharides (lcFOS) (9:1), 0.3% human milk oligosaccharides (HMOS), or a combination (GFH) for 14 days prior to and during vaccination. Delayed-type hypersensitivity (DTH) was measured by assessing ear swelling following an intradermal challenge. Influvac-specific IgG1 and IgG2a levels were assessed using ELISAs, while splenic T and B lymphocytes were analyzed for frequency and activation via flow cytometry. Additionally, cytokine production was evaluated using murine splenocytes co-cultured with influenza-loaded dendritic cells. Mice on the GFH diet showed a significantly enhanced DTH response (*p* < 0.05), increased serological IgG1 levels, and a significant rise in memory B lymphocytes (CD27+ B220+ CD19+). GFH-fed mice also exhibited more activated splenic Th1 cells (CD69+ CXCR3+ CD4+) and higher IFN-γ production after ex vivo restimulation (*p* < 0.05). These findings suggest that GOS/lcFOS and HMOS, particularly in combination, enhance vaccine responses by improving memory B cells, IgG production, and Th1 cell activation, supporting the potential use of these prebiotics in infant formula for better early-life immune development.

## 1. Introduction

Early-life vaccination is crucial to protect newborns from infectious diseases. However, compared to adults, infants exhibit weaker vaccination responses. This may be partly explained by the interference of pre-existing maternal antibodies, derived from placental transfer during gestation and from breastmilk during lactation [1,2], as well as differences in neonatal innate and adaptive immune responses [3,4,5]. The newborn’s immune system mainly relies on innate immune responses and the activation of naïve lymphocytes due to limited exposure to a diverse range of antigens during the prenatal period [6]. In addition, cellular-based immunity is restricted during early-life, primarily directed toward T helper 2 type immune response instead of T helper 1, resulting in a limited cellular response to infection and vaccination in neonates [3,7,8]. Besides cellular immune responses, humoral immunity is vital for the downstream mechanism of efficient vaccination or response to infections [9]. Antibody titers or serological responses are among the important criteria for assessing the efficacy of vaccines [10], where B cells play a crucial role in producing antibodies. Therefore, for effective early-life vaccination, the proper functioning of humoral and cellular immune response with essential roles of B and T cells is required. Vaccination effectiveness is influenced by different factors such as infectious agents (e.g., genetic variation), vaccine-related factors (dose, route of administration, and adjuvant), nutrition, and gut microbiota [11]. Early-life nutrition might interfere with vaccination efficacy via an effect on microbiota composition due to the close connection between the gut and the immune system [12]. The gastrointestinal microbiota influence both humoral and cellular immune responses to vaccination in different ways [13,14]. For instance, Toll-like receptor 5-mediated sensing of the intestinal microbiota affects the antibody response in influenza vaccination [15]. Prebiotics, such as non-digestible oligosaccharides (NDOs), improve the host microbiome by selectively stimulating beneficial bacteria, which is followed by an improved immune response to antigens, including vaccines [16]. Prenatal exposure to prebiotics affects the ontogeny of B cells in the intestine via modulating the expression of B cell-associated genes, like CD25 in regulatory B cells, resulting in enhanced immune imprinting of tolerogenic B cells in the embryo [17]. Among the early-life nutritional compounds, human milk oligosaccharides (HMOS) play a pivotal immunomodulatory role due to their abundance in human milk. HMOS have diverse neonatal effects, including modulating the gut microbiome, improving the intestinal barrier, anti-infective properties, and boosting the immune system [18,19,20]. The positive effects of HMOS during early-life are mainly attributes to their consumption by specific beneficial bacteria in the infant’s gut microbiota, which not only improves bacterial growth, but also results in production of HMOS metabolites, like short-chain fatty acids (SCFAs). Furthermore, HMOS can restrict the binding of pathogens to the intestinal epithelium and subsequently decrease the infection rate of pathogens [21]. Apart from the microbiome-mediated impact of nutrition on vaccination efficacy, nutritional compounds can also directly exert immunomodulatory effects. For instance, using a germ-free mice model, HMOS microbiome-independently promoted the immunoregulatory function by regulating the genes involved in cellular and inflammatory pathways [22]. Furthermore, sialyllactose and galacto-oligosaccharides (GOS) promote the epithelial barrier functioning in vitro independently of the microbiome [21]. In addition to HMOS, GOS and long-chain fructo-oligosaccharides (lcFOS) can improve the human microbiome by enhancing the *Bifidobacteria* as one of the essential commensal bacteria in the human gut [23]. GOS and lcFOS are commonly used in a 9:1 ratio, which is based on the varied structure of NDOs in human milk [24]. GOS have the ability to support mucosal immunity by promoting Th1 immune responses [25] as an important factor in proper cellular-based immune response for vaccination. In the current study, we aimed to investigate whether the combination of GOS/lcFOS (9:1) with HMOS (GFH) can enhance vaccination efficacy during the critical period of early-life immune system maturation, as infants typically show weaker vaccination responses compared to adults. Using a murine influenza vaccination model, immunological effects were evaluated to identify any potential mechanism on cellular and humoral immune responses.

## 2. Materials and Methods

### 2.1. Mice

Six-week-old female C57BL/6JOlaHsd mice were purchased from Envigo (Horst, The Netherlands) and housed at constant temperature (21 ± 2 °C) and humidity (40–60%) in a 12:12 h light/dark cycle (lights on from 7.00 a.m. to 7.00 p.m.), with ad libitum access to food and tap water, in the animal facility of Utrecht University. All experimental procedures were conducted during the light cycle, and repeated measurements were consistently performed within the same time block each day. Animals were group-housed (3 mice/cage) in Makrolon cages (22 cm × 16 cm × 14 cm, floor area 350 cm^2^, Tecnilab-BMI, Someren, The Netherlands) with fine aspen wood-chip bedding (Tecnilab-BMI, Someren, The Netherlands). Plastic shelters (Tecnilab-BMI, Someren, The Netherlands) and tissues (VWR, Amsterdam, The Netherlands) were available as cage enrichment. After a week of acclimatization, one set of mice was assigned to the donor-mice group. During the study, these mice were used for bone marrow isolation from which dendritic cells derived from bone marrow were cultured for ex vivo antigen restimulation experiments. Another set of mice was assigned to the sham-treated group, which was included to demonstrate the specificity of vaccine-induced responses. The remaining mice were randomly assigned to the experimental groups without any inclusion/exclusion criteria. Furthermore, cage handling/shelving was also performed randomly. Animal care and use were conducted according to the principles of good laboratory animal care in compliance with the European Directive 2010/63/EU for the protection of animals used for scientific purposes. All experimental procedures followed the ethical guidelines for animal research established by Utrecht University and the Central Authority for Scientific Procedures on Animals (CCD). The study protocols were reviewed and approved by local Animal Welfare Body at Utrecht University with approval number of 460-1-06 (13 January 2021).

### 2.2. Diet

Semi-purified AIN-93G soy protein based diet [26] was prepared and used as the control diet. The same diet containing the 0.7% GOS/lcFOS (9:1 ratio), 0.3% HMOS, or the combination of 0.7% GOS/lcFOS + 0.3% HMOS (GFH) (Ssniff Spezialdiaten GmbH Soest, Germany) was used to test the effects of GOS/lcFOS and HMOS individually, as well as in combination (GFH), on vaccine-induced immune responses. Vivinal^®^ GOS syrup (∼45% GOS with a degree of polymerization (DP) of 2–8, 16% free lactose, 14% glucose, and 25% water) [27,28,29] was obtained from Friesland Campina Domo, Amersfoort, The Netherlands. lcFOS, long-chain inulin, Raftiline HP (≥97% purity, average DP of 23 or higher) was provided by Orafti (Wijchen, The Netherlands) [30]. The HMOS mix (96.2%), including 2′-fucosyllactose (37.4%), 3-fucosyllactose (22.4%), 3′-sialyllactose (9.2%), and 6′-sialyllactose (27.2%), was biotechnologically produced and obtained from Danone Global Research and Innovation Center B.V. (Utrecht, The Netherlands). The proportions of GOS/lcFOS and HMOS were substituted with equivalent amounts (*wt*/*wt*) of total carbohydrates in the control diet. No significant difference in weight gain was observed between vaccinated mice consuming a diet with GOS/lcFOS, HMOS, or their combination (GFH) and those in the control group (Supplementary Figure S1).

### 2.3. Vaccination Protocol

**Figure 1:**
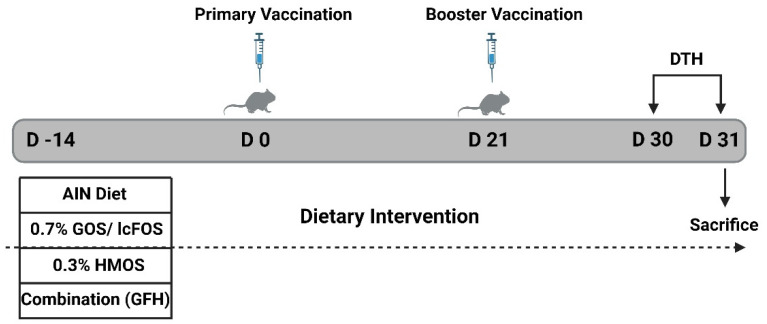
Schematic overview of the study design. D: day, DTH: delayed-type hypersensitivity, GOS: galacto-oligosaccharides, lcFOS: long-chain fructo-oligosaccharides, HMOS: human milk oligosaccharides.

The dietary intervention started two weeks before the primary vaccination, on the animals’ arrival day (day −14). Mice were given the control diet (AIN93G), or control diet supplemented with 0.7% GOS/lcFOS (9:1 ratio), 0.3% HMOS, or their combination (GFH) until the end of the experiment (day 31) (Figure 1). Mice received a primary vaccination on day 0 using influvac (Abbott Biologicals B.V., Weesp, The Netherlands) from season 2019/2020 by subcutaneous (s.c.) injection of 125 μL of vaccine as depicted in Figure 1. influvac is an inactivated influenza virus vaccine that uses isolated hemagglutinin (HA) and neuraminidase antigens from three strains of influenza virus, with a dose of 30 μg/mL HA per strain resulting in a total of 90 μg/mL HA. A booster vaccination was administered 21 days after the primary vaccination. Sham-treated (control) mice received s.c. injections of 125 μL PBS instead of influvac. Nine days after the booster vaccination, antigen-specific delayed-type hypersensitivity (DTH) was induced on day 30 and measured based on ear swelling under deep isoflurane anesthesia after 24 h (day 31).

### 2.4. Antigen-Specific Delayed-Type Hypersensitivity

Delayed-type hypersensitivity response (DTH) was initiated on experimental day 30 and measured using an already described method [31]. Briefly, 20 μL of influvac was injected intradermally (i.d.) into the pinna of the right ear, and 20 μL of PBS was injected into the left ear to establish a baseline for ear swelling. Ear thickness, which serves as an indicator of T helper 1-dependent cellular immunity, was measured twice prior to the antigen challenge at time 0 h (day 30), and 24 h afterward on experimental day 31, using a digital micrometer (Mitutoyo Digimatic). The DTH response was determined by using the formula below, in which the basal ear thickness was subtracted from the measurement taken 24 h after the challenge, correcting for the ear swelling resulting from the i.d. injection of PBS: ΔDTH (µm) = (ear thickness at 24 h)—(ear thickness at 0 h) and ΔΔDTH (µm) = ΔDTH of right ear—ΔDTH of left ear. Following the intradermal challenge, blood samples were collected through orbital extraction under isoflurane anesthesia, after which the animals were sacrificed by cervical dislocation. Hereafter, tissue samples were collected for ex vivo analyses.

### 2.5. Vaccine-Specific Immunoglobulin (Ig) Analysis in Serum

To analyse the impact of dietary intervention on serum concentrations of influvac-specific IgG1/IgG2a, the obtained blood samples were centrifuged (12,298× *g* for 10 min) and sera were collected and stored at −70 °C until subsequent analysis using Enzyme-Linked Immunosorbent Assays (ELISAs) as described previously [32]. Briefly, the antibodies were measured using highly-binding 96-well plates (Costar EIA/RIA plate, Alphen a/d Rijn, The Netherlands) coated with diluted influvac. After blocking with Bovine Serum Albumin (BSA), serum dilutions were added to the wells, followed by anti-IgG1-biotin or anti-IgG2a-biotin antibodies (Becton Dickinson, Heerhugowaard, The Netherlands), and streptavidin-HRP (Biosource, Etten-Leur, The Netherlands) incubations in the next steps. The HRP enzyme activity was visualized using Tetra Methyl Benzidine substrate (1-Step^TM^ Ultra TMB-ELISA, Thermo Fisher, Waltham, MA, USA), and the optical density was assessed at a wavelength of 450 nm using a microplate reader (Promega Corporation, Madison, WI, USA). The dilution factors for IgG1 and IgG2a were optimized as 4000× and 60,000×, respectively. All steps were performed at room temperature. The results are presented as arbitrary units (AU), with pooled sera used as positive references to create a titration curve.

### 2.6. Splenocytes Isolation

Single-cell suspensions from freshly isolated splenocytes were obtained using a previously described method [33]. Briefly, the spleen samples were smashed through the nylon mesh filters (Falcon cell strainer; Becton Dickinson, Alphen a/d Rijn, The Netherlands). Red blood cells were removed by incubating the cells in a lysis buffer (8.3 g NH_4_Cl, 1 g KHC_3_O, and 37.2 mg EDTA dissolved in 1 L demi water and filter sterilized). Hereafter splenocytes were counted and resuspended in RPMI 1640 medium supplemented with 10% fetal bovine serum and penicillin (100 U/mL)/streptomycin (100 µg/mL) for flowcytometric and restimulation analysis.

### 2.7. Flow Cytometric Analysis of Splenocytes

Freshly isolated splenocytes were washed in PBS containing 1% BSA and then incubated with anti-mouse CD16/CD32 (1:100; Mouse BD Fc Block, BD Pharmingen, San Jose, CA, USA). The complete list of used antibodies for splenocyte staining are shown in Supplementary Table S1. Viable cells were identified using a fixable viability dye eFluor^®^ 780 (eBioscience, Thermo Fisher Scientific, San Diego, CA, USA). Briefly, splenocytes were stained using fluorochrome-conjugated monoclonal antibodies against CD3-PerCP-Cy5.5, CD4-PerCp-Cy5.5, and CD25-PerCP-Cy5.5 (BioLegend, San Diego, CA, USA), CXCR3-PE, CD69-PE-Cy7, B220-FITC, CD19-APC, and CD27-PE (eBiosciences, Thermo Fisher Scientific, San Diego, CA, USA), and T1/ST2-FITC (MD Biosciences, St. Paul, MN, USA). To detect intracellular transcription factors, cells were initially fixed and permeabilized using the Foxp3 Staining Buffer Set (eBiosciences, Thermo Fisher Scientific, San Diego, CA, USA) according to the manufacturer’s protocol and then stained with RorγT- Alexafluor 647 (BD Pharmingen, San Jose, CA, USA) antibodies. Results were obtained using a BD FACSCanto II flow cytometer (Becton Dickinson, Franklin Lakes, NJ, USA) and analyzed via FlowLogic software 8.7 (Inivai Technologies, Mentone, VIC, Australia).

### 2.8. Development of Bone Marrow-Derived Dendritic Cells

Bone marrow was extracted from the femurs and tibias of 6 healthy untreated mice to obtain immature bone marrow-derived dendritic cells (iBMDCs). Isolated cells were cultured for six days in RPMI 1640 medium (Gibco, Invitrogen, Carlsbad, CA, USA) containing 10% FBS, 10 mM HEPES, 1 mM sodium pyruvate, 100 U/mL penicillin/streptomycin, and Eagle’s minimum essential medium non-essential amino acids (Gibco, Invitrogen, Carlsbad, CA, USA) and supplemented with 10 ng/mL granulocyte-macrophage colony-stimulating factor (GMCSF) (Prosepec, Zoeterwoude, The Netherlands) [31]. To obtain matured DCs, iBMDCs were incubated with the influvac vaccine at a concentration of 0.9 µg/mL for 24 h at 37 °C in a 5% CO_2_ environment.

### 2.9. Splenocyte Restimulation Using Vaccine-Loaded BMDCs Ex Vivo

Freshly isolated splenocytes were co-cultured with matured BMDCs in the ratio of 10:1, in a 96-well U-bottom plate for five days at 37 °C and 5% CO_2_. Using a ProcartaPlex multiple protein assay kit (Invitrogen), the cytokine concentrations for IL-5, IL-2, IL-6, IL-10, IL-13, IFN-γ,IL-21, IL-27, IL-33, and IL-12p70 in the supernatants of the co-culture were analyzed according to manufacturer’s instructions.

### 2.10. Statistical Analysis

G*Power v3.1.9 was used to calculate required sample sizes, based on DTH measurements from previous experiments [31]. A 10% change in ear thickness was considered to be a clinically relevant difference. The vaccinated groups consisted of n = 9 animals each. The statistical power was set at 0.9 and the significance level (α) was adjusted for the number of relevant comparisons (α/k, k = 5). Sham-treated mice were used to validate the model, therefore a group size of n = 3 was regarded as sufficient. All the obtained results were analyzed via GraphPad Prism 8.0 (Version 8.0, GraphPad, San Diego, CA, USA). Normality of the data sets was assessed via the Shapiro–Wilk test and multiple group comparisons were conducted by one-way ANOVAs followed by a Bonferroni multiple comparison post hoc test. Some samples/data were excluded from the analysis due to technical errors during the assays, resulting in statistical analysis lower than the calculated sample size (n < 9). All results are shown as mean ± SEM and differences were considered statistically significant at *p* < 0.05 (* *p* < 0.05, ** *p* < 0.01, *** *p* < 0.001).

## 3. Results

### 3.1. The Combination of GOS/lcFOS and HMOS Enhances the Delayed-Type Hypersensitivity Response

**Figure 2:**
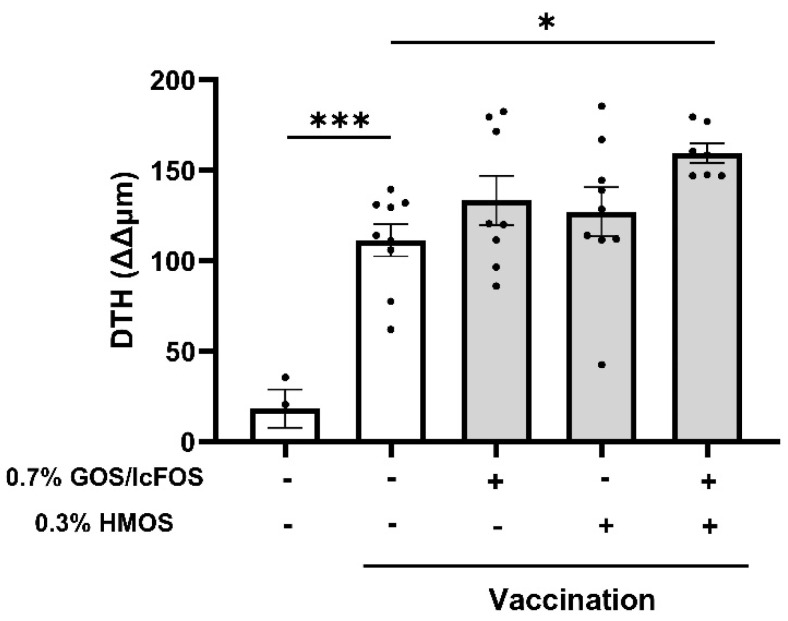
Effect of dietary GOS/lcFOS, HMOS, or their combination (GFH) on influvac-specific delayed-type hypersensitivity (DTH) response. DTH (ΔΔ) representative of DTH corrected for the ear swelling resulting from the intradermal injection of PBS into the ear. Results are shown based on mean ± SEM. * *p* < 0.05 and *** *p* < 0.001. Sham control: n = 3, positive control: n = 9, 0.7% GOS/lcFOS: n = 8, 0.3% HMOS: n = 9, and 0.7% GOS/lcFOS + 0.3% HMOS: n = 7.

Analysis of the ear thickness measurements after influvac injection demonstrated a significant increase in DTH values in the vaccinated mice compared to the sham-treated controls (Figure 2). Compared to the vaccination group, no significant changes in DTH were detected in vaccinated mice that received diets containing either 0.7% GOS/lcFOS or 0.3% HMOS alone. However, the ear thickness was significantly higher in mice given the diet containing the GFH (*p* < 0.05).

### 3.2. Influvac-Specific Immunoglobulins Are Increased after Oligosaccharide Intervention

**Figure 3:**
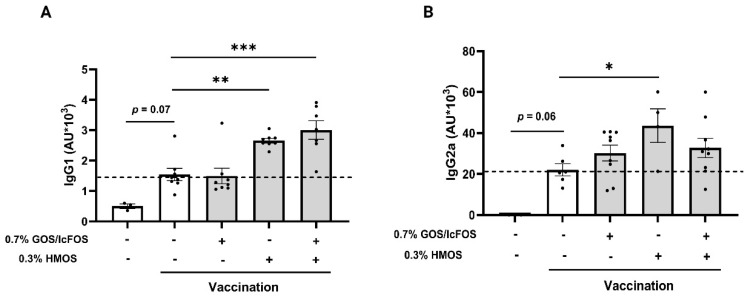
Effect of dietary GOS/lcFOS, HMOS, or their combination (GFH) on serum concentration of influvac-specific IgG1 and IgG2a. Vaccine-specific (**A**) IgG1 and (**B**) IgG2a levels in sera of experimental groups measured by ELISAs. Data are presented as mean ± SEM. * *p* < 0.05, ** *p* < 0.01, and *** *p* < 0.001. (**A**) Sham control: n = 3, positive control: n = 9, 0.7% GOS/lcFOS: n = 8, 0.3% HMOS: n = 8, and 0.7% GOS/lcFOS + 0.3% HMOS: n = 7. (**B**) Sham control: n = 3, positive control: n = 6, 0.7% GOS/lcFOS: n = 9, 0.3% HMOS: n = 4, and 0.7% GOS/lcFOS + 0.3% HMOS: n = 9.

All vaccinated mice showed higher sera levels of influvac-specific IgG1 and IgG2a compared to the non-vaccinated group. Compared to the vaccination group fed a control diet, no significant changes were observed in influvac-specific IgG1 and IgG2a in vaccinated mice fed a diet containing 0.7% GOS/lcFOS alone. However, in vaccinated mice fed a diet with 0.3% HMOS alone or in combination with 0.7% GOS/lcFOS (GFH), serum concentrations of IgG1 were significantly higher (*p* < 0.001) (Figure 3A). Vaccinated mice that received 0.3% HMOS showed significantly (*p* < 0.05) increased serum IgG2a concentration levels as compared to the vaccination group fed a control diet. However, the combined intervention GFH did not significantly affect the serum IgG2a levels (Figure 3B).

### 3.3. Dietary HMOS and GOS/lcFOS Enhances Splenic Memory B Cells

**Figure 4:**
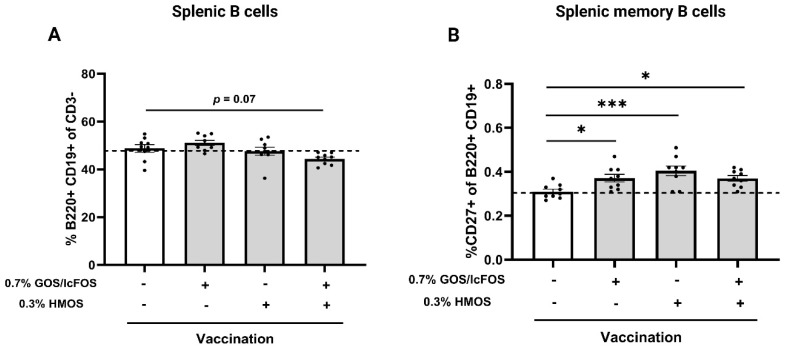
Effect of dietary GOS/lcFOS, HMOS, or their combination (GFH) on splenic B and memory B cells in vaccinated mice. (**A**) Percentage of splenic B cells (B220+ CD19+ of CD3− cells) among experimental groups and (**B**) percentage of splenic memory B cells (CD27+ of B220+ CD19+) among experimental groups assessed by flow cytometry. Data are shown based on mean ± SEM. * *p* < 0.05 and *** *p* < 0.001. n = 9.

The splenic B cells (B220+ CD19+ of CD3− cells) and memory B cells (CD27+ of B220+ CD19+ cells) were analyzed using flow cytometry. The gating strategy for analyzing the splenic B cells and memory B cells is depicted in Supplementary Figure S2. CD3 as an effective T cell marker was used to exclude the T cells from analysis. No significant differences in the frequency of B220+ CD19+ splenic B cells were detected in the dietary intervention groups compared to the positive control (vaccinated) group (Figure 4A). To assess the impact of oligosaccharides on memory B cells, the expression of CD27+ in B220+ CD19+ splenic B cells was analyzed (Figure 4B). Compared to the vaccination group fed the control diet, a significant increase in the frequency of CD27+ in B220+ CD19+ B cells was observed in vaccinated mice receiving diets containing 0.7% GOS/lcFOS (*p* < 0.05), 0.3% HMOS (*p* < 0.01), or the GFH (*p* < 0.05).

### 3.4. The Combination of GOS/lcFOS and HMOS Increases Activation of Splenic T Helper 1 Cells

**Figure 5:**
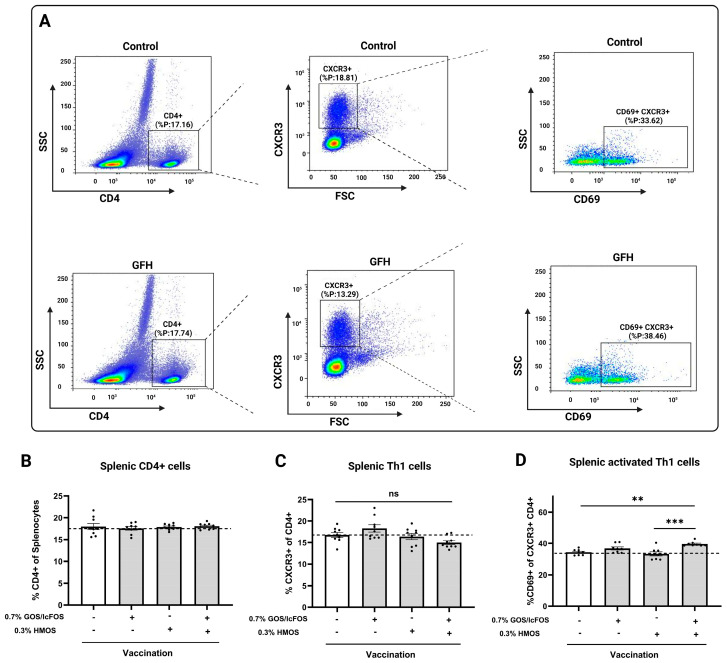
Effect of dietary GOS/lcFOS, HMOS, or their combination (GFH) on frequency and activation status of splenic Th1 cells in vaccinated mice. (**A**) Representative dot plots of splenic Th1 cells in vaccinated (control) mice with and without GFH intervention assessed by flow cytometry, (**B**) percentage of CD4+ cells of splenocytes, (**C**) percentage of CXCR3+ (Th1) cells of CD4+ cells, and (**D**) percentage of activated splenic Th1 cells (CD69+ of CXCR3+ CD4+ cells). Data are shown based on mean ± SEM. ** *p* < 0.01 and *** *p* < 0.001, ns = non-significant. n = 9.

The flow cytometric gating strategy for detecting splenic activated Th1 cells in control and GFH-fed mice is shown in Figure 5A. As depicted in Figure 5B,C, no significant difference was detected in the frequency of either splenic CD4+ or CXCR3+ of CD4+ cells among the groups. Using CD69 staining as an indicator of T lymphocyte activation, vaccinated mice that received GOS/lcFOS or HMOS did not indicate any effect on the activation stage of splenic Th1 cells. Vaccinated mice that received the combined intervention of GFH showed a significant increase in the activation of splenic Th1 cells (CD69+ of CXCR3+ CD4+ cells) as compared to vaccinated mice fed a control diet (*p* < 0.01) (Figure 5D). No significant differences were observed among the experimental groups in the frequency of T1/ST2+ CD4+ Th2 cells, CD69+ T1ST2+ CD4+ activated Th2 cells, CD25+ CD4+ regulatory T cells, and RorγT+ CD4+ Th17 cells (Supplementary Figures S3 and S4).

### 3.5. The Combination of GOS/lcFOS and HMOS Increases the Production of IFN-γ in Splenocytes-DCs when Co-Cultured Ex Vivo

**Figure 6:**
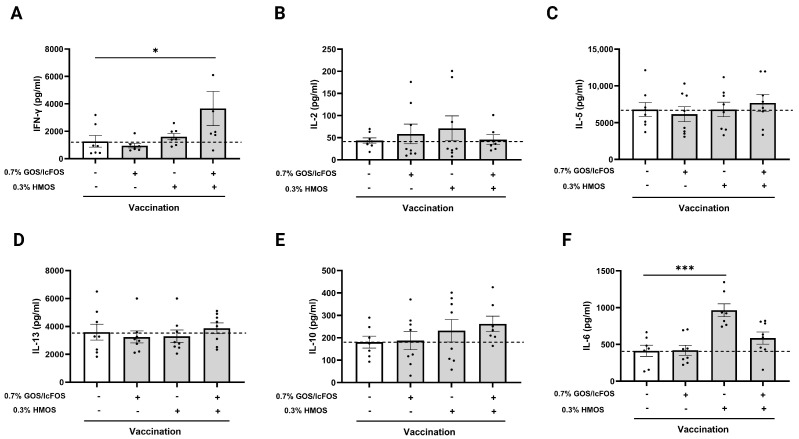
Vaccine-specific cytokine production of splenocytes-DCs co-culture using influenza-loaded bone marrow-derived dendritic cells (BMDCs). Influenza-loaded BMDCs were co-cultured with splenocytes (at a ratio of 1:10 for 5 days) from vaccinated mice with and without a dietary GOS/lcFOS, HMOS, or the combination (GFH) intervention. Co-culture supernatants were collected on day 5 and different cytokines were measured. (**A**) IFN-γ and (**B**) interleukin (IL)-2 as Th1-related cytokines, (**C**) IL-5 and (**D**) IL-13 as Th2-related cytokines, (**E**) IL-10 as Treg-related anti-inflammatory cytokine, and (**F**) pro-inflammatory IL-6. All the cytokines were measured by a ProcartaPlex multiple protein assay. Data are presented as mean ± SEM. * *p* < 0.05 and *** *p* < 0.001. (**A**) Positive control: n = 8, 0.7% GOS/lcFOS: n = 8, 0.3% HMOS: n = 8, and 0.7% GOS/lcFOS + 0.3% HMOS: n = 8, (**B**) Positive control: n = 9, 0.7% GOS/lcFOS: n = 9, 0.3% HMOS: n = 9, and 0.7% GOS/lcFOS + 0.3% HMOS: n = 8, (**C**) n = 9, (**D**) n = 9, (**E**) Positive control: n = 8, 0.7% GOS/lcFOS: n = 9, 0.3% HMOS: n = 9, and 0.7% GOS/lcFOS + 0.3% HMOS: n = 8, and (**F**) Positive control: n = 8, 0.7% GOS/lcFOS: n = 9, 0.3% HMOS: n = 8, and 0.7% GOS/lcFOS + 0.3% HMOS: n = 9.

As shown in Figure 6A, the splenocytes from vaccinated mice fed the GFH co-cultured with influenza-loaded bone marrow-derived dendritic cells (BMDCs) showed a significant increase in interferon (IFN)-γ production after ex vivo restimulation compared to the vaccinated control group (*p* < 0.05). No significant difference among the experimental groups was observed in interleukin (IL)-2 production (Figure 6B); a cytokine associated with Th1 response. GOS/lcFOS and HMOS intervention also did not affect the IL-5 and IL-13 production; cytokines associated with Th2 response (Figure 6C,D). In addition, Treg-related IL-10 anti-inflammatory cytokine production remained unchanged by GOS/lcFOS and HMOS intervention (Figure 6E). Despite the increasing effect of HMOS on IL-6 production in co-culture (*p* < 0.001), GFH did not induce any changes in IL-6 production (Figure 6F) in vaccinated mice. IL-21, IL-27, IL-33, and IL-12p70 were not detectable in the co-culture supernatant.

## 4. Discussion

In the present study, we used a murine influenza vaccination model to assess the potential beneficial effects of GOS/lcFOS, HMOS, or their combination (GFH) on the induced vaccination response. Human milk is rich in prebiotic oligosaccharides, providing the most optimal early-life nutrition with health benefits like immune protection. Nevertheless, due to various circumstances, infant formula feeding is an alternative worth considering. GOS and lcFOS are commonly used oligosaccharides in infant formula where HMOS are absent, meaning the infants on this formula are deprived of the benefits of HMOS. Hence, their combination (GFH) was selected as the dietary intervention approach for this vaccination model.

Vaccination is an established method to evaluate the (Th1) response of the immune system to a specific antigen such as influenza, and it presents a promising opportunity for studying the immunomodulatory effects of different substances including nutritional compounds [34] and early-life risk factors like mycotoxins [32] and antibiotics [35].

Early-life vaccination is crucial to develop robust immune protection, yet a challenge arises when administering vaccines at early-life. Infant immune response tends to favor Th2 responses over Th1 responses. This early-life Th2-oriented cellular immune response is complicated by various exposome factors, like antibiotics and dietary factors, that not only contribute to immune responses tending towards Th2 responses but also diminish the vaccination efficacy. For instance, antibiotics adversely affect immunogenicity and responses to influenza vaccinations in humans [36], which intensifies the challenge of low vaccination responsiveness, meaning that fully vaccinated infants remain vulnerable to potentially life-threatening infectious diseases [37]. Vaccine-specific serological concentrations of immunoglobulins is strongly correlated with vaccination efficacy in infants [38], and both frequency and activation status of B cells play a role in vaccination efficacy [39]. Therefore, enhancing B cells might improve the vaccination efficacy via improved IgG production. Our findings indicated that GFH enhances the DTH response and serum IgG1 concentrations in vaccinated mice, which corresponds with elevated levels of CD27+ memory B cells.

A variety of exposomes, including nutrition, can impact the immunogenicity of vaccines and the following serum antibody concentration. Based on a meta-analysis for clinical trials, it is known that dietary fibers (prebiotics) can affect the serum concentration of immunoglobulins [40]. Prebiotics promote the expansion of B cells, both locally and systemically, that can contribute to the overall development of the immune system by up-regulating immunoglobulin secretion [17]. GOS/lcFOS, a common prebiotic mixture in infant formula, can induce a favorable antibody profile in cow’s milk protein (CMP) allergy by modulating the immune response toward CMP, while leaving the vaccination responses unaffected [41]. Despite our previous findings showing a stimulatory effect of GOS/lcFOS on DTH, we could not detect any GOS/lcFOS-mediated effect on either the DTH values or serum IgG levels. This can be partially explained by the lower administrated concentration of GOS/lcFOS compared to the prior study.

Furthermore, HMOS, as the third most abundant solid constituent in human milk [42], can induce various early-life effects, including immunomodulation, protection of the intestinal barrier, and promotion of neurocognitive function [43]. Here, we demonstrated that both HMOS and GOS/lcFOS, when administrated alone or in combination (GFH), improve the splenic memory B cells frequency without any effect on naïve splenic B cells. In line with the current study, the combination of GOS/lcFOS and 2′FL enhanced the CD27+ mesenteric lymph node memory B cells in the same murine vaccination model [44]. The enhanced frequency of splenic memory B cells by GOS/lcFOS, HMOS, and their combination (GFH) in vaccinated mice was associated with increased serological levels of IgG1 and IgG2a only in the vaccinated mice that received the intervention with HMOS or the GFH. The booster effect of HMOS on splenic memory B cells and the subsequent increased secretion of IgG1 and IgG2a might be partially due to its increasing effect on splenocyte capacity for IL-6 production. IL-6 can enhance the immune response via promoting immunoglobulin production [45]. This primarily speculates that HMOS may play a larger role in improving humoral immunity, as dietary intervention with GOS/lcFOS alone could not enhance the vaccine-specific antibody responses in the current study either. Our speculation gains more strength, as 2′FL, the most abundant HMO in human milk [46], has already been reported to have a booster effect on both splenic memory B cells and serological IgG level in the same influenza vaccination model [31]. A previous study conducted within our research group showed that dietary 2′FL (1%) not only increases splenic B cells (CD19+ B220+) and memory B cells (CD27+ CD19+ B220+) in vivo [31], but also promotes Th1 responses in vitro [47], shedding light on the immunomodulatory effects of 2′FL. Despite the improving effect of 2′FL on splenic B cells (CD19+ B220+), in the present study, HMOS (0.3%) alone or in combination with GOS/lcFOS (0.7%) could not enhance the splenic B cells frequency. Moreover, no significant differences were observed between DTH responses of vaccinated mice fed control diets and vaccinated mice fed a diet containing either GOS/lcFOS or HMOS, while mice that received the GFH demonstrated elevated DTH responses. In agreement with our study, GOS/lcFOS alone was not effective in other vaccine-specific antibody responses related to diphtheria, whooping cough, poliomyelitis [48], tetanus, hemophilus influenza type b, and hepatitis B [49].

It is already known that Th1 plays a critical role in DTH responses via IFN-γ secretion; a cytokine that stimulates macrophages [50]. Our analysis showed that the frequency of activated splenic Th1 cells (CD69+ CXCR3+ cells) was significantly higher in the vaccinated group that received the GFH without any effect on the frequency of splenic Th1 cells, indicating a possible additive effect of the GFH on splenic Th1 activation compared to GOS/lcFOS or HMOS alone. Furthermore, splenocytes of vaccinated mice fed the GFH showed an improved IFN-γ production ex vivo when restimulated with influvac-loaded BMDCs. These results can partially explain the effect of the GFH diet on the DTH response. Despite the demonstrated effect of the GFH on splenic Th1 activation and ex vivo IFN-γ production, the frequency and activation of Th2 cells and the related cytokines remained unaffected by dietary intervention by the GFH (Supplementary Figure S3).

Enhanced Th1 responsiveness due to an intervention with a combination of GOS/lcFOS/pectin-derived acidic oligosaccharides and probiotic can bolster immunity against viral infections, which can improve immune functioning during early infancy [51,52]. As demonstrated in our previous study, the combination of GOS/lcFOS and 2′FL can improve the immune responsiveness by modulating the gut microbiota and metabolic functions like cecal butyric and propionic acid level [44]. In addition, the modulatory effects of dietary oligosaccharides, like GOS/lcFOS and different HMOS, on microbiome composition and related fermented products, i.e., SCFA, have been previously studied [53,54,55,56,57,58,59,60,61]. Therefore, we speculate that the probable modulatory effect of this specific GFH mixture on gut microbial composition might contribute to improved vaccination efficacy. This aspect was not investigated in our study and can be noted as a limitation of the study. Furthermore, evaluating the combined intervention with GOS/lcFOS and HMOS at a higher concentration is warranted, as it may lead to more pronounced effects than those observed in this study.

## 5. Conclusions

In conclusion, we demonstrated the promising enhancing effect of the combined dietary intervention with GOS/lcFOS and HMOS (GFH) on murine influvac-induced DTH responses, associated serum antibody titers, and splenic memory B cells. Furthermore, GFH increased the splenic Th1 activation and ex vivo IFN-γ production. These results suggest that the combination of GOS/lcFOS and HMOS in infant formula can provide further immunological support in early-life and may even be immuno-protective in elderly populations. Lastly, the observed effects of the GFH need to be validated in clinical settings before being applied to humans.

## Data Availability

The raw data supporting the conclusions of this article will be made available by the authors on request.

## Supplementary Materials

The following supporting information can be downloaded at: https://www.mdpi.com/article/10.3390/nu16172858/s1, Table S1: Antibody list for flow cytometry analysis, Figure S1: Effect of dietary GOS/lcFOS, HMOS, or their combination (GFH) on weight gain in vaccinated mice, Figure S2: Gating strategy for detecting the splenic B cells (B220+ CD19+) and memory B cells (CD27+ of B220+ CD19+), Figure S3: Effect of dietary GOS/lcFOS, HMOS, and their combination (GFH) on the frequency and activation status of splenic Th2 cells in vaccinated mice, Figure S4: Effect of dietary GOS/lcFOS, HMOS, and their combination (GFH) on frequency of splenic Treg and Th17 cells in vaccinated mice.
