# Supplementary material for: Human Milk Oligosaccharides in Combination with Galacto- and Long-Chain Fructo-Oligosaccharides Enhance Vaccination Efficacy in a Murine Influenza Vaccination Model
Source: Nutrients. 2024 Aug 26;16(17):2858. doi: 10.3390/nu16172858 (PMC11397401; doi:10.3390/nu16172858)
Supplement: Supplementary file 1 [file nutrients-16-02858-s001.zip › nutrients-3164462-supplementary.pdf]

## Supplementary Materials

**Table S1.** Antibody list for flow cytometry analysis.

| Marker | Label | Detection filter | Catalog number | Supplier | Dilution |
|---|---|---|---|---|---|
| Viability | eFluor780 | 780/60 | 65-0865-14 | eBiosciences | 1:1100 |
| Fc receptor (CD16/CD32) | - | - | 567021 | BD Pharmingen | 1:100 |
| **T cell markers** | | | | | |
| CD3 | PerCp-Cy5.5 | 695/40 | 100217 | BioLegend | 1:100 |
| CD4 | BV510 | 525/50 | 100553 | Biolegend | 1:160 |
| CXCR3 | PE | 576/26 | 12-1831-82 | eBiosciences | 1:100 |
| T1/ST2 | FITC | 525/50 | 101001F | MD Biosciences | 1:200 |
| CD69 | PE | 576/26 | 12-0691-82 | eBiosciences | 1:640 |
| Foxp3 | FITC | 525/50 | 11-5773 | Thermo Fisher | 1:100 |
| RorγT | Alexa fluor 647 | 660/20 | 562682 | BD Pharmingen | 1:400 |
| CD25 | PerCp-Cy5.5 | 695/40 | 101911 | BioLegend | 1:1280 |
| **B cell markers** | | | | | |
| CD19 | APC | 660/20 | 17-0193-82 | eBiosciences | 1:75 |
| B220 | FITC | 525/50 | 11-0452-82 | eBiosciences | 1:75 |
| CD27 | PE | 576/26 | 12-0271-82 | eBiosciences | 1:400 |

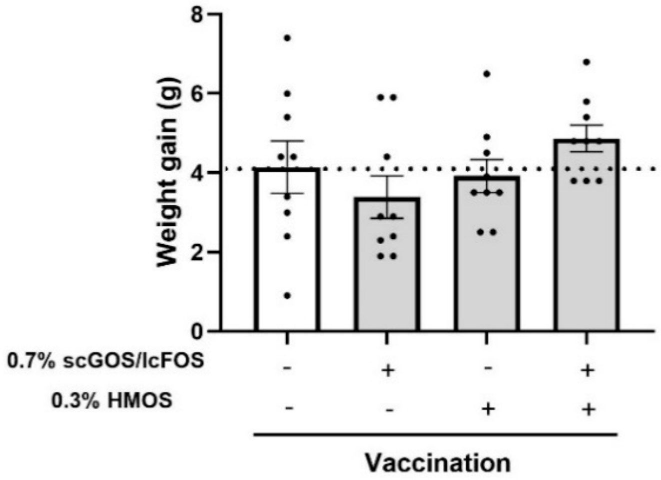


**Supplementary Figure S1.** Effect of dietary GOS/lcFOS, HMOS, or combination (GFH) on weight gain in vaccinated mice. Data are shown based on mean ± SEM. n=9.

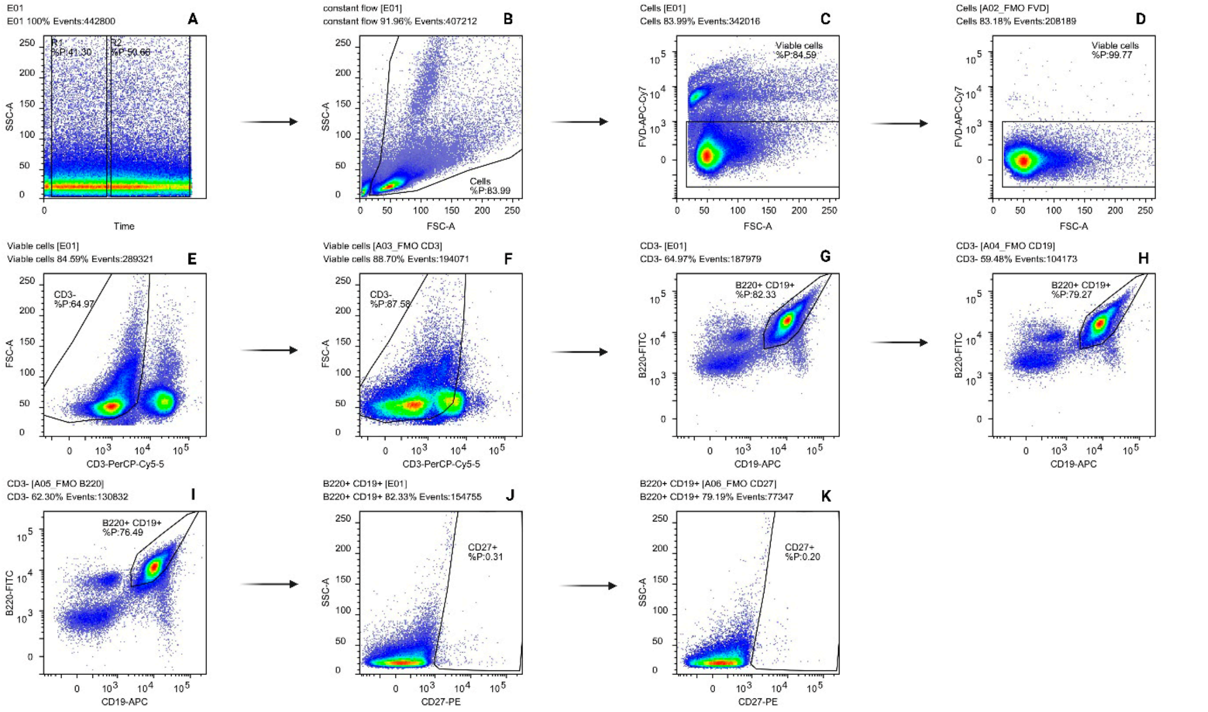


**Supplementary Figure S2.** Gating strategy for detecting the splenic B cells (B220+ CD19+) and memory B cells (CD27+ of B220+ CD19+). **(A)** Constant flow of all events **(B)** Excluding the debris from analysis **(C)** Excluding the dead cells (FVD stained) and continue the gating with live cells **(D)** setting the base line for alive cells based on fluorescence minus one (FMO) control **(E)** Excluding the T lymphocytes from analysis via selecting the CD3- cells **(F)** setting the FMO control for selected CD3- cells **(G)** selecting the B cells via B220 and CD19 marker from CD3- cell population **(H)** setting the FMO control for gated CD19+ B cells **(I)** setting the FMO control for the gated B220+ B cells **(J)** selecting the memory B cells via CD27 marker **(K)** setting the FMO control for gated memory B cells (CD27+).

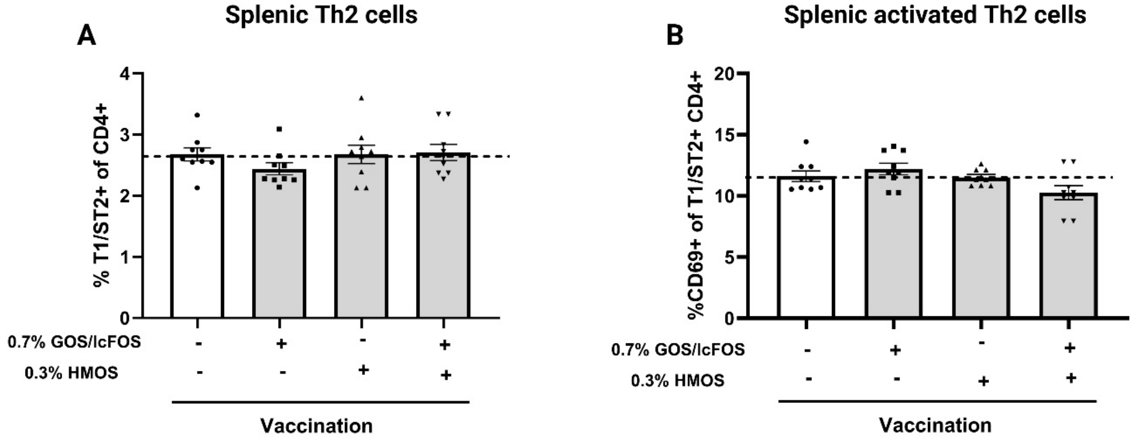


**Supplementary Figure S3.** Effect of dietary GOS/lcFOS, HMOS, and combination (GFH) on frequency and activation status of splenic Th2 cells in vaccinated mice. **(A)** Percentage of T1/ST2+ (Th2) cells of CD4+ cells **(B)** percentage of activated splenic Th2 cells (CD69+ of T1/ST2+ CD4+ cells). Data are shown based on mean ± SEM. n=9.

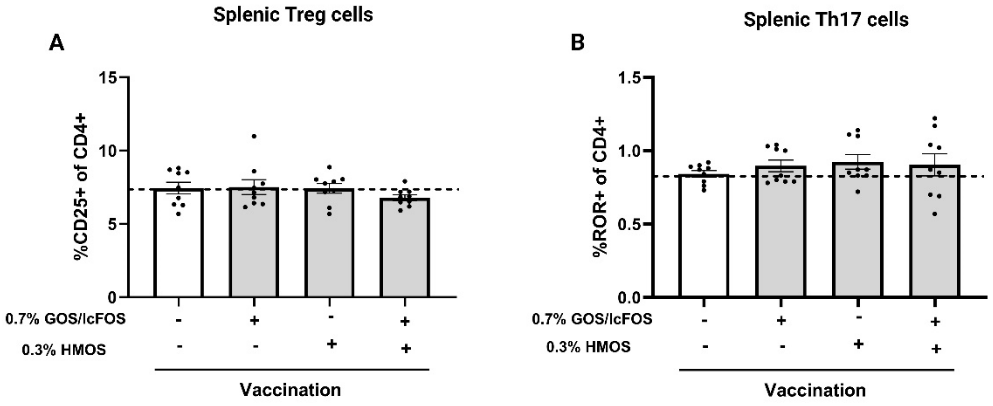


**Supplementary Figure S4.** Effect of dietary GOS/lcFOS, HMOS, and combination (GFH) on frequency of splenic Treg and Th17 cells in vaccinated mice. **(A)** Percentage of CD25+ (Treg) cells of CD4+ cells **(B)** Percentage of Th17 cells (ROR+ of CD4+ cells). Data are shown based on mean ± SEM. n=9.
